# Blood urea nitrogen to serum albumin ratio: a good predictor of in-hospital and 90-day all-cause mortality in patients with acute exacerbations of chronic obstructive pulmonary disease

**DOI:** 10.1186/s12890-022-02258-7

**Published:** 2022-12-15

**Authors:** Zixiong Zeng, Xiaocui Ke, Shan Gong, Xin Huang, Qin Liu, Xiaoying Huang, Juan Cheng, Yuqun Li, Liping Wei

**Affiliations:** Department of Respiratory Medicine, Guangdong Provincial Key Laboratory of Major Obstetric Diseases, The Third Affiliated Hospital of Guangzhou Medical University, Guangzhou, Guangdong China

**Keywords:** AECOPD, Blood urea nitrogen to serum albumin ratio, Mortality, Prognosis

## Abstract

**Background:**

Previous studies on acute exacerbation of chronic obstructive pulmonary disease (AECOPD) have found that those who died in hospital had higher blood urea nitrogen levels and a worse nutritional status compared to survivors. However, the association between the blood urea nitrogen to serum albumin ratio (BUN/ALB ratio) and in-hospital and short-term prognosis in patients with AECOPD remains unclear. The aim of this study was to explore the usefulness of BUN/ALB ratio in AECOPD as an objective predictor for in-hospital and 90-day all-cause mortality.

**Methods:**

We recorded the laboratory and clinical data in patients with AECOPD on admission. By drawing the ROC curve for the patients, we obtained the cut-off point for the BUN/ALB ratio for in-hospital death. Multivariate logistic regression was used for analyses of the factors of in-hospital mortality and multivariate Cox regression was used to analyze the factors of 90-day all-cause mortality.

**Results:**

A total of 362 patients were recruited and 319 patients were finally analyzed. Twenty-three patients died during hospitalization and the fatality rate was 7.2%. Furthermore, 14 patients died by the 90-day follow-up. Compared with in-hospital survivors, patients who died in hospital were older (80.78 ± 6.58 vs. 75.09 ± 9.73 years old, *P* = 0.001), had a higher prevalence of congestive heart failure(69.6% vs. 27.4%, *P* < 0.001), had a higher BUN/ALB ratio [0.329 (0.250–0.399) vs. 0.145 (0.111–0.210), *P* < 0.001], had higher neutrophil counts [10.27 (7.21–14.04) vs. 6.58 (4.58–9.04), *P* < 0.001], higher blood urea nitrogen levels [10.86 (7.10–12.25) vs. 5.35 (4.14–7.40), *P* < 0.001], a lower albumin level (32.58 ± 3.72 vs. 36.26 ± 4.53, *P* < 0.001) and a lower lymphocyte count [0.85 (0.58–1.21) vs. 1.22 (0.86–1.72), *P* = 0.001]. The ROC curve showed that the area under the curve (AUC) of BUN/ALB ratio for in-hospital death was 0.87, (95%CI 0.81–0.93, *P* < 0.001), the best cut-off point value to discriminate survivors from non-survivors in hospital was 0.249, the sensitivity was 78.3%, the specificity was 86.5%, and Youden’s index was 0.648. Having a BUN/ALB ratio ≥ 0.249 was an independent risk factor for both in-hospital and 90-day all-cause mortality after adjustment for relative risk (RR; RR = 15.08, 95% CI 3.80–59.78, *P* < 0.001 for a multivariate logistic regression analysis) and hazard ratio (HR; HR = 5.34, 95% CI 1.62–17.57, *P* = 0.006 for a multivariate Cox regression analysis).

**Conclusion:**

An elevated BUN/ALB ratio was a strong and independent predictor of in-hospital and 90-day all-cause mortality in patients with AECOPD.

## Introduction

Chronic obstructive pulmonary disease (COPD), a complex multi-component disease with chronic systemic inflammation, is a common cause of death around the world [1, 2]. Acute exacerbations in COPD (AECOPD) are key events and they negatively influence the health status of patients, rates of hospitalization, readmission, and disease progression [3, 4]. However, effective methods to identify hospitalized patients with AECOPD with poor outcomes are still lacking. It is vital for clinicians to pay more attention to evaluating the severity and prognosis of patients with AECOPD in advance. Some severity scoring systems had been developed to assist with predicting the outcomes for AECOPD, such as the APACHE II score, DECAF score and SAPS II score [5–7]. Although these scoring systems have shown great potential for accurately predicting AECOPD prognoses [6, 8, 9], not all patients cooperate with clinicians’ investigations; for example, patients with dementia or the elderly. In addition, the subjectivity of some clinicians may affect the scoring system [10].

Based on previous studies, many blood biomarkers have been reported to predict the prognoses and indicate the severity of AECOPD, such as C-reactive protein and procalcitonin [11, 12]. Previous studies have found that non-survivors had higher blood urea nitrogen (BUN) levels and poorer nutritional status than survivors [13–15]. Therefore, we hypothesized that patients with AECOPD with a higher BUN/ALB ratio have an inverse relationship with poorer outcomes of COPD exacerbation. However, to our knowledge, there is no study that has explored the association between BUN/ALB ratio and in-hospital mortality and the short-term prognosis in patients with AECOPD. Accordingly, the objective of our study was to ascertain the prognostic role of BUN/ALB ratio for in-hospital and 90-day all-cause mortality in AECOPD.

## Methods

### Study design and participants

According to the Global Initiative for Chronic Obstructive Lung Disease (GOLD) criteria [16], patients who were diagnosed with COPD and admitted to the respiratory medicine department of the Third Affiliated Hospital of Guangzhou Medical University (Guangzhou, People’s Republic of China) from January 6, 2016 to October 11, 2021 were all consecutively recruited. Only the first admission was included in the study. The primary diagnosis was AECOPD (defined as suffering increased respiratory symptoms that required hospitalization) [16] and was made by respiratory physicians. Patients diagnosed with asthma, interstitial lung disease, lung cancer, active pulmonary tuberculosis, and other lung diseases, hepatic diseases, or malignancy were not included in the study. However, we did not exclude patients with AECOPD with chronic kidney disease or bronchiectasis. Patients without spirometry data were also excluded. All patients provided written informed consent. When patients were discharged, they were followed up every one month for 90 days by telephone. The ethics committee of the Third Affiliated Hospital of Guangzhou Medical University approved the research proposal (No.E202207130425) and this study was conducted in accordance with the Declaration of Helsinki.

### Data collection and definitions

Through our patients’ electronic medical records, demographic (age, sex, body mass index, and smoking history) and clinical (the number of exacerbations in the previous year and comorbidities) data were collected. From hospitalized AECOPD patients’ blood samples, arterial blood gases (PH, PaO_2_, and PaCO_2_), routine blood tests (neutrophil count and lymphocyte count), BUN, and serum albumin were measured. On the basis of our previous article [13], diagnoses of renal dysfunction and congestive heart failure were not described in detail again in this study. A glomerular filtration rate (GFR) < 90 mL/min/1.73m^2^ was considered as renal dysfunction. Based on FEV1, we divided the GOLD stage into GOLD four groups (GOLD 1: FEV1 ≥ 80% predicted; GOLD 2: 50% ≤ FEV1 < 80% predicted; GOLD 3: 30% ≤ FEV1 < 50% predicted; GOLD 4: FEV1 < 30% predicted) [16].

### Statistical analysis

In this study, the primary outcome was in-hospital and 90-day all-cause mortality. Another outcome was the factors associated with in-hospital and 90-day all-cause mortality. Normally distributed variables were expressed as the mean ± standard deviation and non-normally distributed variables were expressed as medians (interquartile range, IQR). Differences between the two groups were tested with a two-independent samples *t*-test and Mann–Whitney U-test, respectively. Categorical variables were expressed as percentages and were analyzed using a Chi-squared test. A receiver operator curve was used to determine the threshold for BUN/ALB ratio. Logistic regression was used for analyses of the factors of in-hospital mortality. The variables detected in the univariate analyses (with a *p* value of ≤ 0.2) were included in the multivariate analyses [17], while controlling for relevant covariates (age, sex, smoking status, BMI, GOLD stage, heart failure and renal dysfunction). Univariate and multivariate Cox regression were used for analyses of the relationship between BUN/ALB ratio and 90-day mortality. Based on the optimal cut-off value, we divided the whole cohort of patients with AECOPD into two groups. The Kaplan–Meier estimator and log-rank test were used to reveal the difference in 90-day mortality between the two groups. In order to limit the influence of CHF and RD on BUN/ALB ratio, we performed a subgroup analysis. Three models were used to clarify the relationship between BUN/ALB ratio and 90-day mortality. Model 1 only included BUN/ALB ratio. Model 2 controlled for baseline covariates (age, BMI, sex and smoking status). Based on Model 2, we constructed Model 3 with the addition of comorbidities and clinical variables that were previously reported and related to COPD mortality (GOLD stage, exacerbations during the preceding year, and PaCO_2_) [18–20]. All analyses were two-sided and *P* < 0. 05 was considered to indicate statistical significance. Statistical analyses were performed with SPSS 17.0 for windows (SPSS, Inc., Chicago, IL, USA).

## Results

### BUN/ALB ratio and in-hospital mortality

**Fig. 1:**
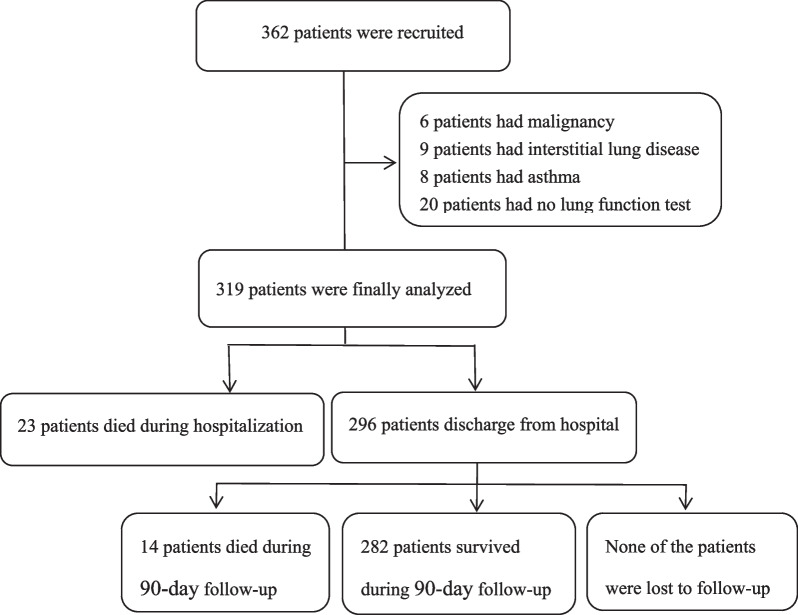
Flow chart of study patients

A total of 362 patients were recruited in this study and 319 patients with AECOPD were finally enrolled and followed up (Fig. 1). During hospitalization, 23 patients died and the differences between the survivor group and non-survivor group during this period are presented in Table 1. Compared with the survivor group, there was a higher incidence of heart failure in the group of deceased patients (69.6 vs. 27.4%, *P* < 0.001) and exacerbations during the preceding year (65.2 vs. 42.9%, *P* = 0.038). Furthermore, those who died were older (80.78 ± 6.58 vs. 75.09 ± 9.73 years old, *P* = 0.001) than the survivor group and also had a higher neutrophil count [10.27 (7.21–14.04) vs. 6.58 (4.58–9.04) × 10^9^/L, *P* < 0.001)], a lower lymphocyte count [0.85 (0.58–1.21) vs. 1.22 (0.86–1.72) × 10^9^/L, *P* = 0.001)], a higher BUN level [10.86 (7.10–12.25) vs. 5.35 (4.14–7.40), *P* < 0.001)], a lower albumin level (32.58 ± 3.72 vs. 36.26 ± 4.53 g/L, *P* < 0.001), a higher BUN/ALB ratio [0.329 (0.250–0.399) vs. 0.145 (0.111–0.210), *P* < 0.001)] compared with the survivor group. Conversely, there were no significant differences in sex, smoking status, GOLD stage, PH, PaO_2_, PaCO_2_, and the length of hospital stay between the two groups.

**Table 1:** Characteristics of AECOPD patients according to in-hospital mortality

Characteristic	Non-survivors (n = 23)	Survivors (n = 296)	*P* value
Age, years	80.78 ± 6.58	75.09 ± 9.73	0.001
BMI, kg/m^2^	20.07 ± 3.97	21.05 ± 3.89	0.244
Sex, n (%)			0.308
Male	17 (73.9)	244 (82.4)	
Female	6 (26.1)	52 (17.6)	
Smoking status, n (%)			0.920
Never smoker	4 (17.4)	61 (20.6)	
Current/ever smoker	19 (82.6)	235 (79.4)	
Hospital stay, days	7.7 ± 4.6	8.0 ± 3.0	0.795
Exacerbations during preceding year, n (%)	15 (65.2)	127 (42.9)	0.038
GOLD stage, n (%)			0.771
I	1 (4.3)	20 (6.7)	
II	11 (47.8)	110 (37.2)	
III	8 (34.8)	116 (39.2)	
IV	3 (13.0)	50 (16.9)	
CHF, n (%)	16 (69.6)	81 (27.4)	< 0.001
RD, n (%)	10 (43.5)	141 (47.6)	0.701
Bronchiectasis, n (%)	4 (17.4)	91 (30.7)	0.238
Diabetes, n (%)	2 (8.7)	30 (10.1)	0.825
Hypertension, n (%)	13 (56.5)	163 (55.1)	0.893
CHD, n (%)	8 (34.8)	76 (25.7)	0.339
Neutrophil count, × 10^9^/L	10.27 (7.21–14.04)	6.58 (4.58–9.04)	< 0.001
Lymphocyte count, × 10^9^/L	0.85 (0.58–1.21)	1.22 (0.86–1.72)	0.001
PH	7.36 ± 0.15	7.40 ± 0.04	0.262
PaO_2_, mmHg	85.1 ± 33.2	90.0 ± 25.2	0.382
PaCO_2_, mmHg	50.7 ± 23.4	44.7 ± 10.2	0.231
BUN, mmol/L	10.86 (7.10–12.25)	5.35 (4.14–7.40)	< 0.001
ALB, g/L	32.58 ± 3.72	36.26 ± 4.53	< 0.001
BUN/ALB ratio	0.329 (0.250–0.399)	0.145 (0.111–0.210)	< 0.001

Variables are expressed as mean ± standard deviation (SD), medians (interquartile range, IQR) or percentages

*GOLD* Global Initiative for Chronic Obstructive Lung Disease, *CHF* congestive heart failure, *RD* renal dysfunction, *CHD* coronary heart disease, *PaO*_*2*_ arterial oxygen tension, *PaCO*_*2*_ arterial carbon dioxide tension, *BUN* blood urea nitrogen, *ALB *albumin, *BUN/ALB ratio* blood urea nitrogen to serum albumin ratio

### The ROC curve analysis of BUN/ALB ratio, 1/ALB, and BUN for predicting in-hospital mortality

**Fig. 2:**
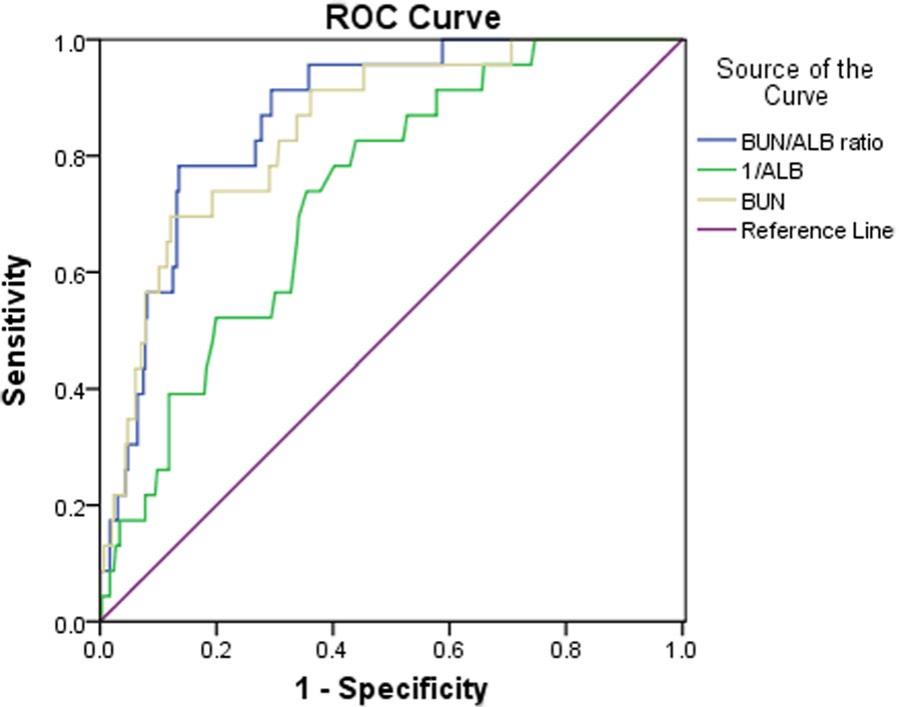
The analysis of receiver-operating characteristics curve for predicting mortality. The area under the curve was 0.87 for BUN/ALB ratio, 0.73 for 1/ALB, and 0.85 for BUN. AUC, area under the curve; CI, confidence interval; BUN/ALB ratio, blood urea nitrogen to serum albumin ratio; ALB, albumin; BUN, blood urea nitrogen

The area under the curve (AUC) of BUN/ALB ratio for in-hospital death was 0.87 (95% CI 0.81–0.93), and the cut-off value was 0.249 with a sensitivity of 78.3% and a specificity of 86.5% (Fig. 2). In addition, the sensitivity and specificity values of 1/ALB and BUN for in-hospital mortality are presented in Table 2. According to the BUN/ALB ratios, we divided the patients into two groups. There were 59 patients with a BUN/ALB ratio ≥ 0.249 and 260 patients with a BUN/ALB ratio < 0.249. As shown in Table 3, there were significant differences between the two groups regarding age, neutrophil count, lymphocyte count, BUN, serum albumin and congestive heart failure, renal dysfunction, hypertension and CHD. However, there were no significant differences between the two groups regarding sex, BMI, smoking status, GOLD stage, exacerbations during the preceding year, PH, PaO_2_, and PaCO_2_.

**Table 2:** Comparison of the ROC curve analysis of BUN/ALB ratio, 1/ALB, and BUN for predicting in-hospital mortality

Parameters	AUC	95% CI	Cut-off value	Sensitivity,%	Specificity,%	Youden’s index	*P* value
BUN/ALB ratio	0.87	0.81–0.93	0.249	78.3	86.5	0.648	< 0.001
1/ALB	0.73	0.64–0.82	0.028	82.6	56.1	0.387	< 0.001
BUN, mmol/L	0.85	0.77–0.92	9.06	69.6	87.8	0.574	< 0.001

*AUC* area under the curve, *CI* confidence interval

**Table 3:** Baseline characteristics stratified by BUN/ALB ratio

Characteristic	BUN/ALB ratio ≥ 0.249 (59)	BUN/ALB ratio < 0.249 (260)	*P* value
Hospitalization mortality (%)	30.5	1.9	< 0.001
Age, years	80.5 ± 8.3	74.4 ± 9.6	< 0.001
BMI, kg/m^2^	20.46 ± 3.83	21.10 ± 3.91	0.257
Sex, n (%)			0.518
Male	50 (84.7)	211 (81.2)	
Female	9 (15.3)	49 (18.8)	
Smoking status, n (%)			0.469
Never smoked	10 (16.9)	55 (21.2)	
Current smoker/Ever smoked	49 (83.1)	205 (78.8)	
Hospital stay, days	8.2 ± 4.2	7.9 ± 2.8	0.472
Exacerbations during preceding year, n (%)	27 (45.8)	115 (44.2)	0.831
GOLD stage, n (%)			0.485
I	3 (5.0)	18 (6.9)	
II	27 (45.8)	94 (36.2)	
III	22 (37.3)	102 (39.2)	
IV	7 (11.9)	46 (17.7)	
CHF, n (%)	26 (44.1)	71 (27.3)	0.012
RD, n (%)	37 (62.7)	114 (43.8)	0.009
Bronchiectasis, n (%)	16 (27.1)	79 (30.4)	0.620
Diabetes, n (%)	6 (10.2)	26 (10.0)	0.969
Hypertension, n (%)	40 (67.8)	136 (52.3)	0.031
CHD, n (%)	22 (37.3)	62 (23.8)	0.034
Neutrophil count, × 10^9^/L	10.27 (7.21–14.04)	6.58 (4.58–9.04)	0.001
Lymphocyte count, × 10^9^/L	0.85 (0.58–1.21)	1.22 (0.86–1.72)	< 0.001
PH	7.38 ± 0.09	7.40 ± 0.05	0.239
PaO_2_, mmHg	90.7 ± 29.0	89.4 ± 25.2	0.732
PaCO_2_, mmHg	44.9 ± 14.3	45.2 ± 11.0	0.870
BUN, mmol/L	10.86 (7.10–12.25)	5.35 (4.14–7.40)	< 0.001
ALB, g/L	32.69 ± 4.36	36.74 ± 4.28	< 0.001

Variables are expressed as mean ± standard deviation (SD), medians (interquartile range, IQR) or percentages.

*CHF* congestive heart failure, *RD* renal dysfunction, *CHD* coronary heart disease, *PaO*_*2*_ arterial oxygen tension, *PaCO*_*2*_ arterial carbon dioxide tension, *BUN* blood urea nitrogen, *ALB* albumin

### BUN/ALB ratio and in-hospital mortality

In a univariate logistic regression analysis (Table 4), we found that age (per increase of 5-year), exacerbations during the preceding year, neutrophil count (per increase of 1 × 10^9^/L), lymphocyte count (per increase of 1 × 10^9^/L), a BUN/ALB ratio of  ≥ 0.249, pH < 7.35, pH > 7.45, and PaO_2_ < 60 mmHg, a history of CHF were risk factors for in-hospital mortality. A BUN/ALB ratio of  ≥ 0.249 was the best predictor for in-hospital mortality (RR = 22.39, 95% CI 7.88–63.62, *P* < 0.001). A multiple logistic regression analysis only indicated that the neutrophil count (per increase of 1 × 10^9^/L), pH < 7.35, a history of CHF, and a BUN/ALB ratio of  ≥ 0.249 were related to in-hospital mortality (Table 4). A BUN/ALB ratio of  ≥ 0.249 still remained a strong predictor of in-hospital mortality (RR = 15.08, 95% CI 3.80–59.78, *P* < 0.001).

**Table 4:** Univariate and multivariate logistic regression analyses of factors affecting in-hospital mortality

Variable	Univariate analysis		Multivariate analysis	
	RR (95% CI)	*P* value	RR (95% CI)	*P* value
Age (per increase of 5-year)	1.47 (1.11–1.96)	0.008	0.95 (0.60–1.51)	0.836
BMI (per increase of 1 point)	0.94 (0.84–1.05)	0.243	0.90 (0.76–1.06)	0.201
Sex (female vs. male)	0.60 (0.23–1.61)	0.312	0.35 (0.05–2.19)	0.259
Smoking status (never smoker vs. current/ever smoker)	1.23 (0.41–3.76)	0.713	3.09 (0.40–23.70)	0.277
Exacerbations during preceding year (yes vs. no)	2.50 (1.03–6.07)	0.044	2.93 (0.74–11.59)	0.127
GOLD stage (per increase to next stage)	0.87 (0.52–1.45)	0.590		
CHF (yes vs. no)	6.07 (2.41–15.29)	< 0.001	4.36 (1.07–17.85)	0.040
RD (yes vs. no)	0.85 (0.36–1.99)	0.701	0.32 (0.09–1.13)	0.076
Bronchiectasis (yes vs. no)	0.47 (0.16–1.43)	0.186		
Diabetes (yes vs. no)	0.84 (0.19–3.78)	0.825		
Hypertension (yes vs. no)	1.06 (0.45–2.50)	0.893		
CHD (yes vs. no)	1.54 (0.63–3.79)	0.343		
Neutrophil count(per increase of 1 × 10^9^/L)	1.16 (1.08–1.24)	< 0.001	1.15 (1.02–1.30)	0.020
Lymphocyte count(per increase of 1 × 10^9^/L)	0.24 (0.09–0.59)	0.002	0.36 (0.09–1.47)	0.154
PH				
(PH < 7.35 vs. 7.35 ≤ PH ≤ 7.45)	5.08 (1.78–14.48)	0.002	8.87 (1.62–48.55)	0.012
(PH > 7.45 vs. 7.35 ≤ PH ≤ 7.45)	7.31 (2.51–21.32)	< 0.001	4.04 (0.87–18.81)	0.075
PaO_2_ (≥ 60 vs. < 60 mmHg)	4.56 (1.51–13.77)	0.007	4.86 (0.89–26.67)	0.069
PaCO_2_ (≥ 50 vs. < 50 mmHg)	1.88 (0.74–4.77)	0.187		
BUN/ALB ratio (≥ 0.249 vs. < 0.249)	22.39 (7.88–63.62)	< 0.001	15.08 (3.80–59.78)	< 0.001

### Survival and risk analyses of the relationship between BUN/ALB ratio and 90-day mortality

**Fig. 3:**
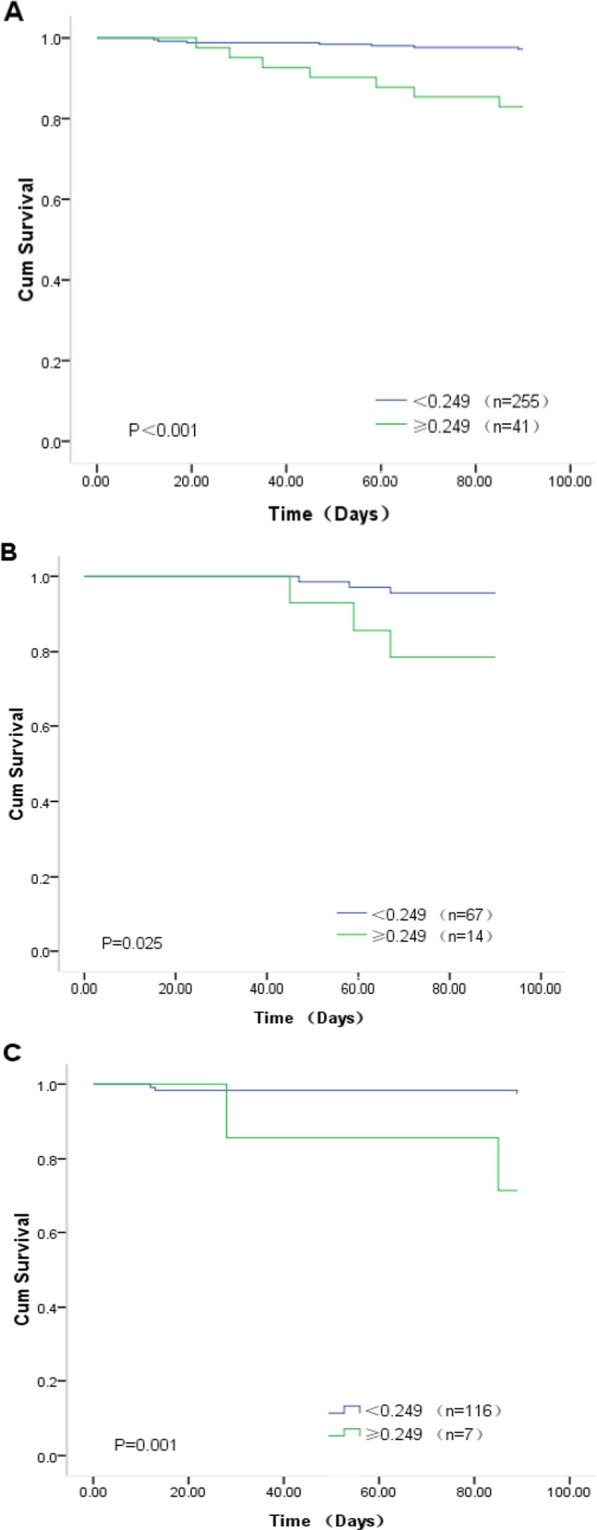
Kaplan–Meier survival curves evaluating the time to death in days for patients. **A** Survival curves of all patients;** B** survival curves of patients with CHF and/or RD; **C** survival curves of patients without CHF and RD

In this study, 14 patients died (4.73%) during follow-up. A Kaplan–Meier estimator and log-rank test were used to reveal the difference between the BUN/ALB ratio of  ≥ 0.249 group and BUN/ALB ratio of  < 0.249 group (Fig. 3A). We found a significant difference between the two groups (*P* < 0.001). In the BUN/ALB ratio of  ≥ 0.249 group, the survival rate was lower than in the BUN/ALB ratio of  < 0.249 group (82.9 vs. 97.25%, *P* < 0.001). In a subgroup analysis, we also observed that there were significant differences in survival curves between the two groups for both the patients with CHF and/or the RD subgroup (survival rate,78.6 vs. 95.5%, *P* = 0.025; Fig. 3B) and the patients without CHF and RD subgroup (survival rate, 71.4 vs. 97.4%, *P* = 0.001; Fig. 3C). Three models were used to clarify the relationship between BUN/ALB ratio and 90-day mortality with the use of multivariate Cox proportional hazards analysis (Table 5). Model 1 showed the unadjusted HR (HR = 6.62, 95% CI 2.32–18.88, *P* < 0.001) for 90-day mortality in the BUN/ALB ratio ≥ 0.249 group. After adjusting for baseline characteristics, the HR was 4.08 (95%CI:1.32–12.58, *P* = 0.014) in Model 2. When we performed further adjustments, including the clinical variables and comorbidities, the BUN/ALB ratio ≥ 0.249 remained significant (HR = 5.34, 95% CI 1.62–17.57, *P* = 0.006).

**Table 5:** The influence of BUN/ALB ratio on 90-day all-cause mortality by multivariate Cox proportional hazards analysis

Variable	Model 1		Model 2		Model 3	
	HR (95% CI)	*P* value	HR (95% CI)	*P* value	HR (95% CI)	*P* value
BUN/ALB ratio (≥ 0.249 vs. < 0.249)	6.62 (2.32–18.88)	< 0.001	4.08 (1.32–12.58)	0.014	5.34 (1.62–17.57)	0.006
Age (per increase of 5-year)			1.71 (1.13–2.59)	0.012	2.30 (1.32–4.01)	0.003
BMI (per increase of 1 point)			0.98 (0.85–1.12)	0.730	1.02 (0.89–1.18)	0.751
Sex (male vs. female)			0.64 (0.18–2.35)	0.505	0.46 (0.10–2.05)	0.305
Smoking status (current/ever smoker vs. never smoker)			0.92 (0.25–3.36)	0.904	1.20 (0.27–5.23)	0.812
Exacerbations during preceding year (yes vs. no)					1.46 (0.48–4.44)	0.509
GOLD stage (per increase to next stage)					3.01 (1.07–8.41)	0.036
PaCO_2_ (≥ 50 vs. < 50 mmHg)					2.03 (0.46–8.92)	0.349
CHF (yes vs. no)					0.83 (0.23–3.00)	0.779
RD (yes vs. no)					0.46 (0.14–1.47)	0.191
Bronchiectasis (yes vs. no)					0.64 (0.16–2.57)	0.533
Diabetes (yes vs. no)					2.05 (0.37–11.29)	0.411
Hypertension (yes vs. no)					0.75 (0.19–2.99)	0.686
CHD (yes vs. no)					2.72 (0.82–9.08)	0.104

## Discussion

Blood urea nitrogen(BUN) is the nitrogen component in urea, the final product of metabolism, which originates from the liver and is excreted by the kidney [21]. Based on previous research findings [13, 14], and compared with survivors, a higher BUN level was found in those who died in hospital. In this study, our results also showed that the non-survivor group had a higher BUN level than the survivor group during hospitalization. Thus, we suggest that elevated BUN levels reflect the severity of the disease. In addition, BUN was shown to have a predictive effect on the prognosis of the disease [22, 23]. One retrospective cohort study from China performed by Chen et al. [24]found that an elevated BUN level was related to hospital mortality in COPD patients with exacerbations for those who presented at the emergency department. Figure 2 shows that BUN levels could predict hospitalization outcomes of the hospitalized patients with AECOPD (AUC = 0.85, 95% CI 0.77–0.92, *P* < 0.001). This was similar to previous findings that indicated elevated BUN levels were associated with increased mortality.

COPD is often combined with digestion and absorption dysfunction and high energy consumption, causing the COPD patients to suffer from malnutrition. Not only malnutrition but also systemic inflammatory responses contribute to a decrease in the albumin levels in serum, leading to a poor prognosis for COPD patients [25, 26]. In our cohort, we observed that those who died during the hospitalization had lower albumin levels than the survivors, which was similar to the results from a previous study [15]. Clinically, we should pay more attention to the nutritional status of patients with AECOPD.

Many studies have reported the prognostic value of BUN/ALB ratio in some respiratory diseases. Ugajin et al. [25]conducted a prospective study with 175 patients and they previously found that an elevated BUN/ALB ratio was a simple but independent predictor of mortality and severity of community-acquired pneumonia. The BUN/ALB ratio could reflect the severity of the patients with pneumonia. For aspiration pneumonia patients, Ryu et al. [27]demonstrated that the BUN/ALB ratio was associated with increased 28-day mortality. In addition, Fang et al. [28]concluded that the BUN/ALB ratio could be a useful prognostic tool to predict mortality in critically ill patients with an acute pulmonary embolism. In our prospective cohort study, we first investigated the association between BUN/ALB ratio and in-hospital and 90-day all-cause mortality. Interestingly, after adjusting the confounding factors, we found that a BUN/ALB ratio ≥ 0.249 was a strong and an independent risk factor of in-hospital (RR = 15.08, 95% CI 3.80–59.78, *P* < 0.001) and 90-day all-cause mortality (HR = 5.34, 95% CI 1.62–17.57, *P* = 0.006) in patients with AECOPD. Therefore, we considered that our study might suggest a supplement to the BUN/ALB ratio as a prognostic factor for respiratory diseases. With stratified BUN/ALB ratios, we observed that those with a high BUN/ALB ratio level had increased in-hospital (30.5 vs. 1.9%, *P* < 0.001) and 90-day all-cause (17.1 vs. 2.7%, *P* < 0.001) mortality. These results indicated that higher BUN/ALB ratios meant patients with AECOPD had higher BUN levels and lower albumin levels in serum. Thus, a higher BUN/ALB ratio often reflects the severity and increased mortality of AECOPD. Many factors might explain the association between a high BUN/ALB ratio level and in-hospital and 90-day mortality in patients with AECOPD. First, once patients with COPD suffer acute exacerbations, they usually suffer from infection [16] and the inflammatory response will accelerate the process of proteolysis and the BUN levels will be elevated due to lower albumin levels in patients. Second, congestive heart failure is a common comorbidity in COPD [16]. Because of decreased cardiac output, the renin angiotensin aldosterone system (RAAS) and sympathetic nervous system are activated. Meanwhile, angiotensin and adrenergic stimulation cause renal vasoconstriction and decreased GFR and renal blood flow. This process can enhance the reabsorption of the urea, causing BUN levels to become elevated [29]. In our cohort, we found that the BUN/ALB ratio of  ≥ 0.249 group had more patients with AECOPD with renal dysfunction than the BUN/ALB ratio of  < 0.249 group (62.7% vs. 43.8%, *P* = 0.009). We suggest that renal dysfunction might lead to urea excretion obstruction. Specifically, reabsorption of the urea will be enhanced. However, in this study, we did not find renal dysfunction was associated with in-hospital mortality in AECOPD (RR = 0.32, 95% CI 0.09–1.13, *P* = 0.076), which was inconsistent with previous studies [30, 31]. We suggest that our small sample size or different statistical methods might be the reasons for the different results.

Given that BUN/ALB ratio was associated with in-hospital and short-term prognosis in patients with AECOPD. We recommend that BUN/ALB ratio should gain prominence in clinical work. How to quickly and accurately assess the severity of AECOPD in patients and give corresponding intervention measures to improve the prognosis and outcome is crucially important. One research from Zemans et al. [32]showed that combined clinical variables might be more highly predictive of outcomes than individual clinical variables in COPD. BUN/ALB ratio, combined with BUN and ALB, might play a better role in predicting the outcomes of patients with AECOPD (Fig. 2). Mathioudakis et al. [33] summarized novel data on the diagnosis, phenotyping, targeted treatment, and prevention of AECOPD and indicated that the development and validation of accurate biomarkers for early characterization of the different types of COPD exacerbations is still a challenge. Biomarkers should be measurable quickly and simple to perform. In this research, we showed that an elevated BUN/ALB ratio was associated with increased mortality in patients with AECOPD. BUN and ALB measurements are easy to obtain, convenient to use, and are not affected by the evaluator's subjectivity, which is more realistic in clinical work. Therefore, we suggest that BUN/ALB ratio might be a promising indicator for assessment of the severity of AECOPD in patients.

In this study, we also found that CHF was an independent risk factor for in-hospital mortality (RR = 4.36, 95% CI 1.07–17.85, *P* = 0.040), which was consistent with previous findings that CHF was a significant and independent risk factor for all-cause mortality in COPD and had an influence on the COPD course [34]. Stratified by the BUN/ALB ratio, compared with the BUN/ALB ratio of  < 0.249 group, there were more patients with congestive heart failure in the BUN/ALB ratio of ≥ 0.249 group (44.1% vs. 27.3%, *P* = 0.012). The pathophysiological mechanism behind the association between elevated BUN/ALB ratios and CHF has been previously described [25, 29]. This might indicate that those combined with CHF have a higher BUN/ALB ratio and more attention should be paid to those with CHF when considering adverse hospitalization outcomes.

Compared with COPD, the inflammation in AECOPD is amplified, causing a poor prognosis [35]. In our study, we found that those died in hospital had a higher neutrophil count level than survivors [10.27 (7.21–14.04) vs. 6.58 (4.58–9.04) × 10^9^/L, *P* < 0.001]. Meanwhile, we also found that a higher neutrophil count was an independent predictor of in-hospital mortality in patients with AECOPD after adjustment in a multivariate logistic regression (RR = 1.15, 95% CI 1.02–1.30, *P* = 0.020). More importantly, from Table 3, in the BUN/ALB ratio ≥ 0.249 group, we observed that the neutrophil counts were higher than those with a BUN/ALB ratio of  < 0.249. This indicated that the systemic inflammatory response contributed to a decrease in the albumin levels in serum and increased the BUN levels [25]. As indicated above, a higher neutrophil count might predict adverse hospital outcomes of patients with AECOPD.

There are several limitations in our study. First, this study was a single-center study with a small sample size, especially a small number of patients who died in hospital. However, to the best of our knowledge, this study was the largest prospective cohort study that we know of that was applied to investigate the prognostic of BUN/ALB ratio in patients with AECOPD. Second, although it was the first study to reveal the relationship between BUN/ALB ratio and in-hospital and 90-day all-cause mortality of patients with AECOPD, there is still a lack of previous studies as references. Meanwhile, in the present study, we did not analyze the effect of therapeutic interventions on BUN levels of patients with AECOPD during hospitalization, which may influence the results. We also did not exclude the patients who had chronic kidney disease. As renal dysfunction is common in AECOPD, its inclusion in the cohort might have made our study closer to a real-world study. Third, patients with AECOPD often have severe hypoxia on admission and they may have been given oxygen treatment in the outpatient department before the first blood draw. To some extent, it may influenced the results of our study. Fourth, previous studies [25, 27] have suggested that BUN/ALB ratio was associated with pneumonia. However, clinically, it is difficult to accurately distinguish AECOPD from COPD combined with community-acquired pneumonia, although we only recruited the patients discharged from the hospital with AECOPD as the primary reason for hospitalization. Finally, in our study, we only explored the short-term prognosis and we do not know the association between BUN/ALB ratio and long-term mortality. Thus, the results of this study still need to be further verified by large-sample, multicenter prospective studies in the future.

## Conclusion

In this cohort study, we found that an elevated BUN/ALB ratio was associated with in-hospital and 90-day all-cause mortality in patients with AECOPD.

## Data Availability

Datasets relevant to the study are available from the corresponding author upon reasonable request.

## Abbreviations

GOLD: Global Initiative for Chronic Obstructive Lung Disease
CHF: Congestive heart failure
RD: Renal dysfunction
CHD: Coronary heart disease
PaO_2_: Arterial oxygen tension
PaCO_2_: Arterial carbon dioxide tension
BUN: Blood urea nitrogen
ALB: Albumin
BUN/ALB ratio: Blood urea nitrogen to serum albumin ratio
AUC: Area under the curve
HR: Hazard ratio
95% CI: 95% confidence interval
